# Lactate as a Metabolite and a Regulator in the Central Nervous System

**DOI:** 10.3390/ijms17091450

**Published:** 2016-09-01

**Authors:** Patrizia Proia, Carlo Maria Di Liegro, Gabriella Schiera, Anna Fricano, Italia Di Liegro

**Affiliations:** 1 Department of Psychological, Pedagogical and Educational Sciences, Sport and Exercise Sciences Research Unit, University of Palermo, Palermo I-90128, Italy; patrizia.proia@unipa.it; 2 Department of Biological Chemical and Pharmaceutical Sciences and Technologies (STEBICEF), University of Palermo (UNIPA), Palermo I-90128, Italy; carlomaria.diliegro@unipa.it (C.M.D.L.); gabriella.schiera@unipa.it (G.S.); anna.fricano@unipa.it (A.F.); 3 Department of Experimental Biomedicine and Clinical Neurosciences (BIONEC), University of Palermo, Palermo I-90127, Italy

**Keywords:** lactic acid, brain metabolism, lactate transporters, blood-brain barrier, lactate receptors, exercise and lactate

## Abstract

More than two hundred years after its discovery, lactate still remains an intriguing molecule. Considered for a long time as a waste product of metabolism and the culprit behind muscular fatigue, it was then recognized as an important fuel for many cells. In particular, in the nervous system, it has been proposed that lactate, released by astrocytes in response to neuronal activation, is taken up by neurons, oxidized to pyruvate and used for synthesizing acetyl-CoA to be used for the tricarboxylic acid cycle. More recently, in addition to this metabolic role, the discovery of a specific receptor prompted a reconsideration of its role, and lactate is now seen as a sort of hormone, even involved in processes as complex as memory formation and neuroprotection. As a matter of fact, exercise offers many benefits for our organisms, and seems to delay brain aging and neurodegeneration. Now, exercise induces the production and release of lactate into the blood which can reach the liver, the heart, and also the brain. Can lactate be a beneficial molecule produced during exercise, and offer neuroprotection? In this review, we summarize what we have known on lactate, discussing the roles that have been attributed to this molecule over time.

## 1. Introduction

Carl Wilhelm Scheele, an 18th century Swedish chemist, also known for his role in the discovery of oxygen, purified many organic acids, among which lactic acid (LA).

About one hundred years later, in their study on the regulation of the blood supply to the brain, Roy and Sherrington reported that cerebral anemia, experimentally obtained by closing the carotid and vertebral arteries, was immediately accompanied by an acid reaction in the cortex; then, after reopening the cerebral vessels, the acidity gradually disappeared. The Authors attributed the observed effects to the formation of (ethylidene) lactic acid [1]. In the following years, it was proposed that LA increased during muscle exercise because of an oxygen debt, determined by the high energy requirement of contraction [2,3,4]. In glycolysis, indeed, glucose is broken down to two molecules of pyruvate; pyruvate then either enters the mitochondria, where it is further oxidized in the tricarboxylic acid (TCA) cycle, or is reversibly reduced by NADH(H^+^)-dependent lactic dehydrogenase (LDH) to lactic acid, in order to regenerate NAD^+^ for glycolysis. Shortage of oxygen, by slowing down mitochondrial oxidative phosphorylation, and hence TCA cycle, causes an increase of lactate production. In order to limit cell acidification, both lactate and protons then exit cells.

By the end of the fifties, it was clear that hypoglycaemia, by limiting the supply to the brain of its preferred metabolic substrate (i.e., glucose), could reduce cell respiration/energy production and hence brain electric activity. It was also clear that, upon electrical stimulation, the metabolic activity of the brain could be increased and, as a consequence, more glucose and oxygen were taken from the blood, with a parallel increase of carbon dioxide and lactate released into the blood [5]. Moreover, electrical stimulation of brain tissue rendered it more susceptible to a fall in glucose level, and the same effect was noticed for the aerobic accumulation of LA [6]. These observations suggested that neuronal activity needs to be coupled to an efficient uptake of oxygen and metabolites from the circulation, and that the blood flow itself should be modulated locally in response to variations of neuronal activity [7]. In the following decades, the possibility emerged that cells in the brain itself, namely astrocytes, can transfer to neurons molecules, such as glucose and/or lactate, to sustain increased energy requirements, both in physiological and pathological conditions. Here, we’ll discuss how ideas on the production and utilization in the brain of LA have changed over time from identification of lactate as a metabolic product of glucose metabolism to acknowledgement of its regulatory functions.

## 2. Exercise, Lactate Production in the Periphery and Fatigue

### 2.1. Exercise and Lactate Production

In muscle cells, during intense exercise, ATP is mainly generated from blood glucose and muscle glycogen to fuel glycolysis. This latter pathway is still considered the fastest, but produces less ATP compared to phosphagen systems and to the cellular respiration (oxidative phosphorylation, OXPHOS) [8]. ATP, the fuel used for contraction, is usually present in cells at a concentration of 8 mmol/kg of wet weight of muscle [9]. On the other hand, accumulation of pyruvate, not used for OXPHOS, should inhibit glycolysis because of shortage of NAD^+^; to overcome this situation, in anaerobic conditions (hypoxia), a large part of pyruvate is reduced to lactate by lactate dehydrogenase enzyme, thus oxidizing NADH(H^+^) back to NAD^+^. Noteworthy, during intense physical activity, lactic acid, that is a strong organic acid, mainly exists, like pyruvate, as an anion at the human body pH values. In other words, due to its pKa (3.86), lactic acid is physiologically deprotonated and exists as lactate and H^+^ [10].

In addition, lactate can be also considered an aerobic metabolite, usable by skeletal muscles and by the heart when the oxygen availability is adequate, and it may contribute to acetyl-CoA formation [11]. A large part of muscle-derived lactate is transported to the liver where it is used to synthesize glucose through gluconeogenesis; glucose then enters the blood flow and comes to the muscles to be used as a metabolite in glycolysis (Cori’s cycle). In the last decade, it has been discovered that adipocytes can also produce lactate under anaerobic circumstances. It has been suggested that, in order to counteract O_2_ shortage, adipocytes switch to glycolytic metabolism, and to avoid a dramatic intracellular pH decrease, protons are removed from the cell thanks to proton-linked monocarboxylate transporters (MCTs; isoforms 1 and 4; see next section) [12]. The total amount of lactate produced by adipocytes depends on the fat mass: it is indeed higher in obese subjects than in normal-weight individuals.

A large part of lactate produced from active muscles is released and taken up by exercising and non-exercising muscles, heart and brain, to be oxidized and used as a fuel [13]. More than 30 years ago, Brooks introduced the concept of lactate shuttle through which lactate might be carried into mitochondria or peroxisomes, through MCTs, to be reoxidized, or even out into the bloodstream to be taken up from other organs which could use it as a fuel [14]. In sports medicine, the highest level of physical effort that the body can sustain without accumulating lactate and hydrogen ions (H^+^) in blood and muscles is called anaerobic threshold or lactate threshold; this threshold also corresponds to the intensity/duration of physical activity that the energy metabolism supports without switching from aerobic to anaerobic. Lactate threshold provides a good fitness level and is considered the best performance predictor. Intense training increases lactate threshold and, finally, increases performance [15].

Hypoxia can be induced by intense exercise as well as by altitude exposure (2300–5700 m); the latter induces skeletal muscle adaptations, such as transport of bicarbonate, hydrogen ions, and lactate, all of which are finely regulated. Oxygen shortage stimulates glycolysis and can increase pyruvate availability. This may be used for further oxidation in mitochondria or to increase lactate production. As a result of hypoxia, there is a higher amount of lactate production for a given workload. Muscular energy comes more from carbohydrates than fatty acids. Altitude-induced hypoxia leads to the lactate paradox, defined as “reduced blood lactate concentrations during submaximal and maximal exercise following acclimatization to high altitude” [16]. An interesting hypothesis is that altitude acclimatization may be due to a reduced “muscle recruitment” probably aimed at “protecting” cerebral oxygenation; in other words, the paradox of lactate, observed in chronic hypoxia, should be due to reduced muscle recruitment imposed by the central nervous system, that reduces the transport of O_2_ to muscles and muscle contraction to protect itself from dangerous hypoxia [16,17].

### 2.2. Fatigue

A parallel interesting matter concerns “fatigue”, a condition of rapid deterioration of contractile function, caused by intense exercise. Keeping ATP levels is essential in carrying out the physiological functions of the body. Muscle fatigue is possibly necessary to avoid that ATP range falls below critical or irreversible levels, and it is logical to assume a causal relationship between anaerobic metabolism and muscle fatigue [18]. The shift from aerobic to anaerobic metabolism is probably responsible for the sudden increase in lactate levels in blood and, even before, at the intracellular level, for metabolic acidosis [19]. Acidosis seems to impair muscle contraction, leading to fatigue and, ultimately, to cessation of exercise. The uncomfortable feelings associated with acidosis has long been associated with the pain (now commonly referred to as delayed onset muscle soreness, or DOMS) of the days after intense exercise. Lactate production through the glycolytic pathway is associated with hydrogen ions (H^+^) production: in particular, two ions are released if glycolysis uses blood glucose, while one ion is released if glycogen is used [20]. Thus, lactate is not necessarily the source of the H^+^ increase, which might indeed derive from glycolysis [21]. Historically, the accumulation of lactic acid in muscles has been suggested as the main cause of muscle fatigue. Lactate and H^+^ ions are produced in muscles during intense exercise, and in humans, the intracellular lactate concentration may reach 30 mM or more. Nevertheless, the intracellular pH only decreases of approximately 0.5 units, due to the body buffering capacity. In these conditions, an increase of extracellular osmolarity should be observed, which causes a water movement out of the muscle fibers, and thereby an increase of the intracellular ionic strength. The overall result should be an inhibitory effect on force production [22,23]. On the other hand, acidification per se seems to have a low effect on the reduced fiber shortening velocity linked to fatigue. Lactic acid production plays indeed a metabolic buffering action rather than determining the onset of acidosis. As a matter of fact, the reaction catalyzed by lactate dehydrogenase uses two electrons and one proton from NADH and a second proton from the cytosol to reduce pyruvate to lactate [24]. In summary, the muscle lactate production is essential for the buffering effect as well as to regenerate NAD^+^, at the same time serving to remove the extra pyruvate [9]. A further mechanism by which intracellular acidosis might induce fatigue is through inhibition of key enzymes of glycogenolysis and glycolysis, such as glycogen phosphorylase and phosphofructokinase, both of which are inhibited by low pH, at least in vitro [25].

Another system responsible for ATP synthesis in working muscles involves the phosphagens. During intense exercise, the main substrate used is creatine phosphate (CrP). The reaction catalyzed by creatine kinase uses protons (H^+^), thus causing a faint alkalization of the muscle cell. The effect of this reaction is to counteract the slight acidosis induced by glycolysis and lactic acid formation [26]. Muscle contraction also causes an increase of phosphate (Pi) levels, which has a direct effect on myofibrils and/or an effect on the pathway of excitation/contraction of muscle cells [27]. Several studies suggest that an elevated muscle [H^+^] as well as high [Pi] could impair muscle function by reducing (i) the transition of miofibrils to high cross-bridged states; (ii) the maximal shortening velocity; (iii) the glycolytic rate; (iv) cross-bridging activation, by competitively inhibiting Ca^2+^ binding to troponin C [4,28,29].

It is not yet clear which effect has the predominant role in fatigue; probably, all the mentioned factors are involved, and play a synergistic effect, thus leading to impaired contraction and muscle strength.

## 3. Lactate Uptake across the Blood-Brain Barrier and Monocarboxylate Carriers (MCTs)

### 3.1. Lactate Can Cross the Blood-Brain Barrier (BBB)

During the eighties, the idea that lactic acid (LA) was only a dead-end waste product of anaerobic metabolism began being challenged by many converging observations [4]. First of all, as mentioned, it was realized that LA can be shuttled both between and within the cells. The most relevant output of LA from cells was bound to sustained exercise. This process was defined by Brooks [14] as the “lactate shuttle”. LA is indeed released from muscles during short-term exercise, and even from muscles at rest, during recovery from short-term exercise, and during long-lasting exercise. However, LA can be taken up from blood into either resting or slowly working muscles [4]. Actually, in the same muscle, glycolytic fibers can produce and release LA which can be, at least in part, taken up by surrounding oxidative fibers and again converted to pyruvate for further aerobic oxidization.

As we will discuss later, a relevant case of intercellular lactate shuttle should be that established among glial cells (especially astrocytes) and neurons, which could be considered one of the many aspects of the glial–neuronal metabolic coupling.

In addition to intercellular lactate shuttling, intra-cellular shuttling has been also suggested. In particular, muscular mitochondria were reported to contain a monocarboxylate transporter (MCT1, see below), able to allow LA entrance, as well as lactic dehydrogenase, which converts lactate back to pyruvate. This reaction also transfers electrons to NAD^+^, thus also shuttling electrons from the cytosol to the mitochondrion [4,30,31].

A further indication that lactate is not only a waste product of anaerobic metabolism, produced in conditions of oxygen shortage, but instead a potentially useful fuel, came from the observation that the blood brain barrier (BBB) can be crossed by lactate. The brain is indeed isolated and protected from compounds circulating in the blood by a layer of brain capillary endothelial cells (BCECs) with peculiar properties [32]. BCECs are sealed together by tight junctions (TJs) which block para-cellular fluxes of molecules. Thus, in order to enter the brain, molecules have to be either soluble in plasma membrane lipids (and not actively extruded by ATP-dependent transporters: ABC transporters), or to be recognized by transporters, able to mediate trans-cellular crossing of the BCEC layer [32,33,34,35]. In addition, to ensure trans-cellular transport, a synergic cooperation between the two membranes of endothelial cells is required [36].

### 3.2. The Monocarboxylate Carriers (MCTs)

Lactate entrance into the brain is mediated by BBB transporters which belong to the monocarboxylate carrier (MCT) family, and also transport other monocarboxylic acids, such as pyruvate and ketone bodies. MCTs form a family of 14 transmembrane proteins [37,38], which belong to the solute carrier family (SLC16), and for many of which endogenous functions and substrates still await to be discovered. Actually, lactate transport across the cell plasma membranes of the body can use either sodium-coupled transporters (SMCTs) or proton-coupled monocarboxylate transporters (MCTs) [13]. In the brain, lactate transport involves MCTs. In particular, four carriers (MCTs 1–4) have been characterized which catalyze bidirectional, electroneutral 1:1 co-transport of protons and monocarboxylic acids [13,37,38,39,40]. All of them can transport lactate, with different affinities, the one with the highest affinity being MCT2 (Km: about 0.7 mM). MCT1 is expressed in a variety of human tissues, including brain, and is involved in brain uptake of lactate across the BBB both in mice and humans [41]. MCT2, first reported to be expressed in astrocytes, has been then demonstrated to be primarily present in neurons, and, more precisely, in postsynaptic elements of glutamatergic synapses, where it has been suggested to provide increased supply of lactate “as energy fuel” during neuronal increased activity [39]. MCT3 is preferentially expressed in the basolateral membrane of the retinal pigment epithelium (RPE), and plays an important role in regulating pH and lactate concentration in the outer retina [42]. Finally, MCT4 (Km: about 35 mM) seems to be restricted to the plasma membrane of astrocytes, where it localizes to both perisynaptic processes and perivascular endfeet [39]. The different localization of the MCTs in the brain suggests that each species might be involved in different aspects of lactate metabolism, possibly involving differences in the preferential direction of inter-cellular lactate transport. The role of these transporters might change under different conditions. It has been reported, for example, that oxygen- and glucose-deprivation (OGD) increases cell death in cultured neurons, and up-regulates expression of MCT4 in astrocytes. If, however, cell cultures are exposed to MCT4- or MCT2-specific siRNAs, thus significantly reducing their expression, neuronal cell death increases, unless lactate is added to the culture medium before OGD [43]. Moreover, expression of MCTs has been found to be perturbed in some pathological conditions, such as epilepsy [41]. The MCTs have different Km for lactate, thus probably contributing to the specific tendency of each cell type in a tissue to transport lactate out of or into the cell. It is, however, to be pointed out that the proton gradient is also important [39].

Interestingly, membrane localization and activity of MCTs also require accessory proteins, such as neuroplastin, basigin and embigin [44,45,46], that chaperone their transport to the membrane as well as their function.

Recently, MCT1, MCT2, and MCT4 have been shown to be expressed also in the peripheral nervous system (PNS), with different cell- and domain-distribution: myelinating Schwann cells (SCs) express both MCT1 and MCT4, but in different compartments, while neurons of the dorsal root ganglion express MCT1. Interestingly, downregulation of MCT1 expression can increase myelinating activity in SCs and decrease neurofilament synthesis in neurons [47]. Moreover, the Authors found that lactate homeostasis participates in the regulation of SC myelinating program [47].

## 4. Glial Cell—Neurons Lactate Shuttle and Brain Energy Metabolism

### 4.1. Lactate Shuttling and Energy Metabolism

In the last two decades it has become increasingly clear that glial cells have more functions and are more dynamic than previously expected. Astrocytes, in particular, constitute with neurons a highly integrated complex in which each astrocyte contacts many neurons, and many astrocytes are coupled to each other through astroglial intercellular networks based on gap junction channels (GJ) [48,49,50], formed by connexins (C × 43 and C × 30) [51,52,53]. Importantly, astroglial networks are involved in several control functions, such as ionic homeostasis and control of cell volume; they seem to finely tune neuronal circuits, by delivering to neurons energy metabolites in neuronal activity-dependent manner [49], as also suggested by their distribution and proximity to excitatory synapses [54]. Moreover, C × 43 hemichannels have been reported to allow diffusion of gliotransmitters (for example, glutamate or ATP) [55,56], thus mediating signaling events which seem to be important also for functions as complex as memory consolidation [57], and recovery after ischemic injury [58]. Formation of an astrocytic network also allows generation of a glucose gradient, from the highest concentrations close to the BBB, where glucose uptake at astrocytic feet is maximal, to the lowest concentrations at the level of neuron-astrocyte contacts. Along this gradient, glucose can rapidly move toward the regions at which neuronal activity and hence metabolic demand are higher. Astrocytes are also able to store glucose as glycogen, and it has been proposed that this glycogen is metabolized and supports neuronal activity in conditions of glucose shortage, triggered by a variety of causes [59,60,61,62,63]. Interestingly, glycogen is located in myelinating Schwann cells in the mouse sciatic nerve which is also able to release lactate, thus suggesting that glycogen plays a similar metabolic role in central and peripheral nervous system [64].

Finally, and most important, since many years ago, the existence of an astrocyte-neuron lactate shuttle (ANLS), which should be particularly active during excitatory neurotransmission, has been suggested [65]. In detail, the hypothesis moved from the observation that glutamate released from glutamatergic axon is taken up by both neurons, and astrocytes which surround the synapse. Glutamate uptake is mediated by a sodium-dependent carrier and triggers an increase of the Na^+^/K^+^-ATPase activity; by consuming ATP this process stimulates glycolysis, glucose utilization, and lactate production [65,66,67,68]. Then, lactate should be delivered to neurons through the MCTs present in both astrocytes and neurons. Finally, neurons should convert lactate to pyruvate and use it for synthesizing acetyl-CoA, and fueling the tricarboxylic acid cycle. At the same time, ATP is used in astrocytes to convert glutamate to glutamine which is released to neurons as well [69].

The central, and most important, point of the ANLS model is the neuronal use of astrocyte-derived lactate instead of glucose. Actually, even before the proposal of the lactate shuttle hypothesis, it had been demonstrated that brain slices in culture were able to maintain normal synaptic function using lactate as the only energy source [70], and that lactate, not glucose, was necessary to neurons during recovery from hypoxic conditions [71,72]. Moreover, as discussed by Schurr [73], the idea that brain tissue could oxidize lactate was even older and dates back to the thirties [74,75,76,77,78].

A further important aspect of the ANLS theory is the idea that lactate can come from glial cells [79,80,81]. There is some debate, however, on the origin of this lactate: as already mentioned, the original theory moved from glutamate recovery from the synapse [7,82]. An alternative proposal was that lactate derives from glycogen stored in astrocytes [83,84]. In contrast, it has also been suggested that it derives from slow oxidation of glutamate released from astrocytes in a calcium-dependent manner, and not from glutamate released during glutamatergic neuronal activation [85]. Further controversy concerns the real importance, under physiological conditions, of lactate released from astrocytes for neuronal metabolism [61,86]. It has been proposed, for example, that glycogenolysis’ main function in astrocytes is to maintain a high concentration of glucose-6-phosphate, thus inhibiting hexokinase and sparing glucose; this latter could be thus diverted to neurons [62]. A fundamental question is which cell type (neurons or glial cells) uses more glucose? Under resting conditions, about one half of glucose seems to be taken up by neurons and one half by astrocytes. However, the energy required by astrocytes accounts for about 10%–15% of total energy needed in the brain, and that required by neurons for about 60% or more. This observation suggests that: (i) astrocytes use glucose mainly for anaerobic glycolysis; and (ii) neurons should use something else besides glucose [87]. Lactate might be the substrate produced in astrocytes and used in neurons, as shown at least in vitro [88,89,90,91,92]. Intriguingly, an opposite flux of metabolites (lactate and aspartate) from neurons to Muller glia has been reported in mouse retinas [93]. In addition, experimental evidence has been reported that, during neuronal activation, both neurons and astrocytes can oxidize glucose as well as lactate, and that considerable lactate production might be neuronal [94,95,96,97,98,99,100]. In agreement with the ANLS hypothesis, it has been found that exposure to metabolic stress induces the rapid release from neurons of tissue-type plasminogen activator (tPA). In this case tPA does not induce cleavage of plasminogen but, instead, AMPK activation and recruitment to the membrane of GLUT-1, in both astrocytes and endothelial cells. Uptake and metabolism of glucose is followed by production and release of lactate, which is then captured by neurons through the MCT-2 [101]. Interestingly, astrocyte-neuron lactate transport has also been suggested to be required for long-term memory formation (see below); in particular, it seems that, during the most intense cognitive activities in the hippocampus, astrocytic glycogenolysis is stimulated and provides lactate, which is then transferred to neurons as an energy surplus when glucose is not sufficient [102,103]. In agreement with this idea, it has been recently reported that, in response to local neuronal activation, lactate is released from astrocytes through a K^+^-stimulated anion channel [104].

On the other hand, other researchers reported that, during neuronal activity in hippocampal slices, glucose is the effective energy substrate for both neurons and astrocytes [105,106]. The mentioned controversies could be at least partially solved if lactate is not only a metabolic substrate but also a signaling molecule (see below).

### 4.2. Glucose Sensing

A particularly interesting topic concerns the ability of the brain to “sense” glucose concentration. This ability primarily involves the hypothalamus, the part of the brain able to organize adaptive responses by modulating different functions, among which hormone production, which in turn regulates food intake and peripheral organ activities. In contrast with other brain regions, hypothalamus (and in particular the arcuate nucleus, ARC) is not completely separated from circulation by the BBB and spaces permeable to ions and nutrients are present, close to the nutrient-sensitive neurons [50,107]. Central glucose sensing, in turn, controls neuronal signaling cascades which regulate peripheral hormone-dependent glucose uptake and metabolism [108].

Glucose-sensitive neurons were discovered about 50 years ago [109,110] and their mechanisms of action have been further characterized by ex vivo electrophysiological studies [50]. Recently, the mechanisms underlying glucose sensitivity have been better clarified with the discovery that astrocytes, and specifically connexins, are also involved [111]. Although the main cerebral forms of glucose transporters are the high-affinity ones (GLUT1 and GLUT3), similarly to pancreatic beta cells, hypothalamus glucose sensitivity relies on GLUT2 glucose transporter and on the ability of hexokinase IV (glucokinase, GK) to phosphorylate glucose, without being inhibited by its end-product, glucose-6-phosphate. GK is expressed in selected neuronal populations of hypothalamus [108,112], and, in addition, it has been reported to form a mitochondrial complex with proteins involved in apoptosis control [113]. On the other hand, GLUT2 is mainly present in hypothalamic astrocytes [114,115], thus suggesting that these cells have an important role in sensing glucose concentration. Interestingly, it has been reported that glucose-sensing neurons can also be activated by lactate [116,117], and one possible view is that they sense, at least in part, not directly glucose, but lactate released by astrocytes [50].

### 4.3. Role of Extracellular Vesicles (EVs) in Cell-to-Cell Communications in the Brain

Finally, a short mention should be made on the molecule exchange among brain cells, which involves extracellular vesicles (EVs), either ectosomes or exosomes. Both neurons [118] and astrocytes [119] in culture can release into the medium extracellular vesicles (EVs) which contain, for example, angiogenic factors [118,119], molecular chaperones [120], and synapsin I [121]. This mode of communication is fundamental for tumor cells, including brain cancer cells, for invasion and migration [122,123,124], but it could also have an important role in normal brain cells. Brain cells have indeed the ability to grow branched cellular processes which explore the environment and establish several contacts with other cell types. Interestingly, in vitro studies showed that synaptic activity can potentiate release of exosomes from neurons [125], and that exosomes can be involved in synaptic plasticity and long-term memory [126]. EVs are also released from oligodendrocytes and it has been reported that these vesicles can contain molecules of metabolic interest, among which lactate [127,128,129].

## 5. Lactate as a Substrate during Exercise and in Memory Processes

A growing body of evidence suggests that mild, but regular, physical activity can be beneficial to neuronal survival and plasticity. This observation is especially important in older people. It is indeed clear that increasing age is associated with decreasing cognitive functions, and increasing risk of dementia. It has been reported for example that the number of deaths from Alzheimer’s disease (AD) increased by 68% from 2000 to 2010, in spite of a drastic reduction of mortality from cardiovascular disease and stroke [130]. In general, brain volume decreases with aging. Cognitive decline has been attributed to changes in both neuronal and non-neuronal populations within the CNS, including deterioration of the blood–brain barrier (BBB) [131]. Physical activity seems to ameliorate cognition processes, and counteract brain aging [132,133]. The cellular events responsible for these effects have been, however, only partially characterized.

As expected, during exercise, brain blood flow increases, and this elevation is due to an increase in cardiac output, but probably also to changes in brain metabolism [134]. For example, brain glycogen stored in astrocytes decreases during prolonged exercise with hypoglycemia, and this effect has been attributed to activation of the transduction pathways triggered by noradrenaline and serotonin [135,136,137]. By using an acute intense exercise model of swimming in rats, Matsui et al. [63] also showed that glycogen decreases also in the absence of hypoglycemia, and that its decrease associates with increased lactate in hippocampus, cerebellum, cortex and brain stem [63]. Since glycogen is essentially stored in astrocytes, it is likely that the increase of lactate is due to astrocytic activity. As mentioned, it has been suggested that astrocyte-neuron lactate transport is also important for memory formation [102,103,138]. Could lactate be the link between exercise and improvement of cognitive functions? One of the factors that link exercise and memory seems to be brain-derived neurotrophic factor (BDNF) [139,140,141]. Animal studies have shown that this neurotrophin is essential for long-term potentiation (LTP) and neuroplasticity [142,143,144]. Moreover, pharmacological blockade of BDNF expression hampers the ability to acquire and retain novel spatial information in rats exposed to exercise [145]. Notably, human studies have confirmed that circulating levels of BDNF are transiently increased with exercise [146,147]. Mild exercise can also stimulate hippocampal neurogenesis [148]. However, the biochemical mechanisms underlying the link between exercise-related peripheral BDNF increases and memory improvements in humans remain to be clarified. Two other growth factors are probably involved in the effects of exercise on neuroplasticity: insulin-like growth factor 1 (IGF-1) [149,150,151] and vascular endothelial growth factor (VEGF) [141]. VEGF could have a direct effect on BBB function and, in turn, on oxygen and glucose uptake to the brain, while, interestingly, both IGF-1 and BDNF seem to stimulate neurogenesis, through a signaling cascade that includes calmodulin-dependent protein kinase II (CAMK-II), an enzyme likely involved in long term potentiation [152,153]. CAMK-II is regulated by calcium ions and calmodulin (CaM). In neurons, CaM also interacts with small IQ domain proteins (SNIQ), such as neuromodulin, neurogranin, and PEP-19 [154,155,156,157]. SNIQ have the potential to modulate the amount/localization of calmodulin which can interact with, and activate CAMK-II. This raises the interesting possibility that IQ domain proteins might provide at least some of the still unknown regulatory links between calcium metabolism in the brain and cognitive functions, in both normal and pathological conditions. For example, CAMK-II might phosphorylate RNA-binding proteins which are part of pre-localized ribonucleoparticles (RNPs); phosphorylation of these proteins should then allow translation of specific mRNAs only at the level of activated synapses, thus participating in their potentiation [158]. Notably, at least one of the small IQ domain-containing proteins, PEP19, has been reported to be able to bind mRNA, in alternative to calmodulin [159].

Coming back to lactate, this compound, possibly supplied to neurons from astrocytic glycogenolysis, seems to be an important substrate for neuronal metabolism and LTP maintenance [141]. In addition, during exercise, lactate originating from muscle metabolism can also be shuttled across the BBB through monocarboxylate transporters (MCTs), to be used by brain cells [39,134,160,161]. Interestingly, increases in peripheral blood lactate levels have recently been associated with increased circulating BDNF [162,163]. The biochemical association between the neurotrophin and lactate production has not yet been clearly understood. However, this observation, together with the putative role of lactate in neuron metabolism under conditions of special energy demand, suggests that lactate can indeed represent the link between exercise and exercise-dependent improvement of cognitive functions. Actually, memory formation and consolidation require energy, especially in conditions of arousal and/or stress, and, as mentioned, this energy seems to mostly derive from astrocytic glycogenolysis. In the hippocampus, exercise leads to a burst of extracellular lactate which remains at high levels for at least 50 min and is completely blocked by inhibition of glycogenolysis, which also blocks long-term memory [102]. An important role in memory processes is played by glutamate, and active uptake of glutamate by astrocytes, as already mentioned, is the basis of the astrocyte-neuron shuttle [138].

Moreover, lactate has been recently reported to potentiate NMDA glutamate receptor-mediated currents, which play a central role in neuronal plasticity and memory processes; in doing that, lactate activates a cascade of molecular events which ends up with stimulation of the expression of synaptic plasticity-related genes, such as Arc, c-Fos, and Zif268 [164].

## 6. Lactate as a Signaling Molecule in the Brain

As discussed above, lactate is probably a molecule with an important metabolic impact in the brain. However, a further role is emerging [165,166]: lactate can indeed also bind a receptor of the G protein coupled receptor (GPRs) family: GPR81, also known as HCA1 (hydroxycarboxylic acid 1) or HCAR1 (hydroxycarboxylic acid receptor 1). This receptor is connected to a G protein (Gi) that inhibits adenylate cyclase, thus causing a decrease of the second messenger cyclic AMP (cAMP). The mRNA encoding GPR81 and the protein itself localize to hippocampus, neocortex and cerebellum. The protein is concentrated in the principal neurons (for example, the pyramidal neurons in the hippocampus), but also in the interneurons of many brain areas [167]. By electron microscopy with immunogold, the highest concentration of the receptor was found in the somatodendritic compartment, mainly on the post-synaptic dendritic spines of excitatory synapses [166]. Immunoreactivity was also found on the brain capillary endothelial cells (BBB) and on the perivascular and perisynaptic astrocytic processes. Localization of GPR81 thus suggests that lactate can have at the same time a metabolic role and a regulatory role in the control of blood flow and synaptic function, acting as an intercellular messenger [168]. Moreover, transduction of a signal which decreases cAMP could represent a feedback mechanism, opposing the effect of catecholamines, which induce glycogen breakdown and production of the lactate itself [169,170]. Possibly, signaling through GPR81 (which requires high concentrations of lactate) may have a special role under stress conditions, such as those induced by ischemia or seizures. Actually, it seems that lactate can contribute to signaling pathways in other ways: for example, lactate released from astrocytes attenuates transporter-mediated uptake of prostaglandin E2, thus increasing its external accumulation and vasodilation [171]. However, vasodilation is useful in hypoxia or, in general, in conditions of energy deficiency, which are the conditions of high lactate production and release. Thanks to vasodilation a higher amount of oxygen and glucose can reach the brain.

Interestingly, a different, putatively excitatory G-protein coupled receptor for lactate, which causes an increase of cAMP, has been described in the Locus Coeruleus, but its significance is still under study [170].

Lactate has also been reported to activate the expression of NDRG3 protein. During hypoxia, low oxygen concentration and high lactate levels induce NDRG3 protein that, in turn, stimulates the Raf-ERK pathway to promote angiogenesis and cell growth [172].

Moreover, Rinholm and colleagues showed that low glucose medium conditions reduce number and myelination activity of cultured oligodendrocytes, and that the addition of lactate can rescue both development and myelinating capacity of the cells [173].

Taken together, these observations indicate that lactate plays a very complex role in brain metabolism and function. This role is possibly different in normal and stress/pathological conditions. Thus a final central question is: are elevated concentrations of lactate in the brain the cause or the consequence of stress/pathological conditions?

## 7. Brain Glucose Metabolism and Elevated Lactate in Pathological Conditions

In many acute as well as chronic pathological conditions, brain glucose metabolism is altered. For example, diabetic patients undergo frequent episodes of hypoglycemia, due to the concomitant effects of the therapy itself and the inability of the organism to elicit the physiological responses to a low concentration of circulating glucose [174]. In these patients, as well as in nondiabetic people, lactate infusions can reduce the response to epinephrine, thus reducing the risk of hypoglycemia, while causing brain lactate uptake [161,174,175,176,177]. Similar observations have been done in rats [178]. Now, as discussed above, beside the use of lactate coming from the circulation as a substrate in the place of glucose, an additional explanation for these observations could be that lactate, by binding to its GPR81 receptor, induces a decrease of cAMP, thus counteracting the effects of epinephrine.

In traumatic brain injury (TBI), an increase of brain glycolysis, partially relying on brain glycogen, is observed immediately after injury, in parallel with a decrement in cerebral oxygen consumption rate [179]. In some patients, an elevation of the pentose phosphate pathway was also observed [180]. In the days following TBI, however, glucose uptake and its use rate decrease. At the same time, many patients show cerebral net lactate uptake [181]. A direct use of lactate in patients with TBI was actually demonstrated by a combination of 13C-labelled microdialysis and high-resolution nuclear magnetic resonance [182,183]. For this reason, lactate is beginning to be considered a good substrate for vascular infusion of patients in the days after TBI; lactate indeed should enter the mitochondria to be oxidized to pyruvate and pyruvate could be then used for fueling the TCA cycle. Alternatively, lactate could be oxidized to pyruvate in the cytoplasm and pyruvate could enter the mitochondria. In both cases, lactate should serve as a “glucose sparing” substrate [179,184].

Notably, on the other hand, some Authors have emphasized the uncoupling of metabolism between neurons and astrocytes after TBI, and the production of an excessive amount of lactate, which can be toxic, in the injured brain (“lactate storm”) [185].

In the case of ischemic insults, many in vitro studies suggest that lactate is the major energy substrate for surviving neurons, and it has been reported that lactate can protect neurons from glutamate-induced neurotoxicity [39,186]. Intracerebroventricular as well as intravenous administration of lactate after transient middle cerebral artery occlusion attenuate lesion size and improve neurologic outcome. These effects can be partially explained by the use of lactate as a metabolic substrate and partially by its binding to GPR81, and activation of a signal transduction pathway in neurons [187,188].

Disturbances of glucose metabolism have also been recognized in the pathogenesis of depression. By studying in a model of prenatal stressed rats (an animal model of depression), the expression of different enzymes involved in glycolysis, pentose phosphate pathway, and TCA cycle, data have been collected which suggest that glycolysis is increased and TCA cycle decreased in the brain of prenatal stressed animals, with a parallel increase of lactate [189].

A significant increase in de novo synthesis of lactate (and glutamine) was also observed in the brain of rats which underwent experimental bile-duct ligation to simulate liver fibrosis and necrosis; these rats are a good animal model of chronic liver disease, as they indeed develop brain edema, and minimal hepatic encephalopathy. On the basis of these results, the Authors suggest that inhibiting lactate synthesis could be a target in the treatment of hepatic encephalopathy [190,191].

The levels of lactate were reported to be high in the cerebrospinal fluid (CSF) of patients with Alzheimer’s Diseases (AD) [192]. Now, β-site APP-cleaving enzyme (BACE1), one of the two enzymes which cleave the amyloid precursor protein (APP) to give beta peptides, has an acidic optimal pH; because of this consideration, the levels of beta peptides have been studied in cultured neuroblastoma cells, treated with lactate. According to these studies, high levels of lactate could be a risk factor in AD amyloidogenesis because of aberrant APP processing leading to increased generation of amyloid peptides and APP aggregates; however, the lactate effect does not seem to be due to pH modifications, but to increased levels of ER chaperones Grp78 and Grp94, which leads to aggresome formation [193].

On the other hand, regular physical activity and exercise are protective against many pathologies, such as cardiovascular diseases, and dementia, including AD [130]. As we have discussed, exercise causes an elevation of circulating lactate and this lactate can reach the brain, by crossing the BBB. Notably, similarly good effects of exercise have been reported in Parkinson disease [194] and Huntington’s Disease [195], as well as in multiple sclerosis [196].

As a final note, high levels of serum lactate have been reported to be non-invasive biomarkers of malignancy for brain tumors [197].

## 8. Conclusions

In conclusion, the idea that lactate is a waste product of the metabolism and a main cause of fatigue, should no longer be considered as an indisputable truth. This molecule, instead, can be shuttled among cells and, inside the cells, among different organelles, thanks to specific monocarboxylate carriers, and seems to be a fuel for many cells, including neurons, in conditions of oxygen shortage.

Most importantly, it can behave as a signal, eliciting in the cells’ responses which counteract stress, allowing at least an attempt of adaptive responses. Interestingly, exercise, which promotes lactate production, has been recently reported to have a positive effect in many physiological as well as pathological conditions, including brain aging and neurodegenerative diseases.

Although more research is required to better understand the real function of lactate produced during exercise, what we have been knowing up to now is exciting and suggests that this molecule could somehow underlie the ancient observation that a healthy (and well exercised) body hosts a healthy mind.
